# Corneal Geometry and Refractive Fluctuations Throughout the Day in Orthokeratology Wearers: A Preliminary Study

**DOI:** 10.3390/vision10030048

**Published:** 2026-07-27

**Authors:** Laura Barberán-Bernardos, Miguel Ángel Ariza-Gracia, David P. Piñero

**Affiliations:** 1 Department of Optics, Pharmacology and Anatomy, University of Alicante, 03690 San Vicente del Raspeig, Alicante, Spain; laura.barberan@ua.es; 2 ARTORG Center for Biomedical Engineering Research, University of Bern, 3008 Bern, Switzerland; miguel.ariza@unibe.ch; 3 Department of Ophthalmology (IMQO-Oftalmar), Vithas Medimar International Hospital, 03016 Alicante, Spain

**Keywords:** orthokeratology, circadian rhythm, corneal topography, pachymetry, myopia, corneal asphericity

## Abstract

Despite the widespread use of orthokeratology (ortho-k), the diurnal behavior of corneal geometry after lens removal remains unclear. This prospective observational study evaluated diurnal variations in corneal geometry, refractive error, and visual performance in ortho-k wearers using global and sector-based corneal analyses. Nineteen eyes from ten ortho-k wearers were examined at five time points over a 10-h period following overnight lens wear. Corneal thickness, anterior and posterior corneal curvature, corneal asphericity, autorefractometry, and visual acuity were assessed. Corneal elevation data were reconstructed using Zernike polynomial expansions, and regional analyses were performed using concentric annular sectors. Diurnal amplitudes were compared with a healthy control cohort evaluated using the same protocol. Corneal thickness progressively decreased throughout the day (*p* < 0.001). Anterior corneal curvature showed progressive steepening (*p* ≤ 0.011), whereas posterior corneal curvature exhibited significant flattening (*p* ≤ 0.018). A progressive myopic refractive shift was also observed, with a spherical equivalent amplitude of −1.03 D (*p* = 0.002). Despite these changes, visual acuity remained stable (*p* = 0.248). Compared with healthy controls, ortho-k wearers showed greater amplitudes of central corneal thickness and anterior corneal curvature changes. These findings indicate that ortho-k may enhance normal diurnal corneal changes while maintaining visual acuity.

## 1. Introduction

Orthokeratology (ortho-k) is a non-surgical method of refractive correction based on the overnight reshaping of the cornea using specially designed rigid gas-permeable contact lenses [1]. Through hydraulic and mechanical forces generated beneath the lens, the anterior corneal surface is temporarily molded into a flatter shape, thereby reducing the refractive error, particularly myopia [2,3]. Adequate overnight wear is essential to achieve and maintain the desired refractive effect. Since these corneal changes are reversible, the cornea progressively returns toward its original shape after lens discontinuation or prolonged periods without lens wear. The full ortho-k effect is typically achieved after approximately one week of treatment, whereas corneal and refractive parameters generally return to baseline values within two weeks after lens discontinuation [4,5].

However, not all patients achieve the same effectiveness with ortho-k treatment. This variability in treatment response may be related to differences in corneal biomechanics, since the ortho-k effect depends on the ability of the cornea to undergo temporary reshaping under the forces generated by the lens. Parameters such as corneal stiffness, hysteresis, and corneal resistance factors may influence the magnitude and stability of the refractive correction achieved. González-Méijome et al. reported a better response to ortho-k treatment in corneas with lower hysteresis and resistance measured with the Ocular Response Analyzer from Reichert [6], while Lam et al. reported that patients with a flatter corneal curvature and lower tangent modulus responded worse to the treatment [7].

Previous studies have suggested that corneal changes induced by ortho-k are not completely stable throughout the day, with the anterior cornea becoming progressively steeper within the first hours after lens removal [8,9]. This gradual regression of the corneal reshaping effect may lead to fluctuations in corneal geometry, refractive correction, and visual quality over time. Understanding these diurnal changes is clinically relevant, since the timing of corneal measurements may influence the interpretation of topographic and refractive outcomes during ortho-k follow-up. Furthermore, characterization of the diurnal stability of corneal reshaping may provide further insight into the mechanisms underlying interindividual variability in treatment response.

Although ortho-k is highly dependent on overnight wear duration and is influenced by the normal diurnal physiological changes of the cornea, its exact effects throughout the day have not been fully characterized. These physiological changes include the natural diurnal variation in corneal thickness and biomechanical properties as seen in healthy patients. Following lens removal in the morning, the ortho-k effect is typically at its maximum due to overnight corneal reshaping. During the day, this therapeutic effect gradually regresses, resulting in a more pronounced treatment effect in the morning compared with the late afternoon. Therefore, the aim of this study was to evaluate preliminarily diurnal changes in corneal geometry, refractive error, and visual performance in ortho-k lens wearers using global and sector-based corneal analysis.

## 2. Materials and Methods

### 2.1. Study Design and Participants

A prospective observational study was conducted at the Optometric Clinic of the University of Alicante (Alicante, Spain), following the ethical standards established in the Declaration of Helsinki. Ethical approval was granted by the University of Alicante Ethics Committee (UA-2023-01-19_2), and all subjects signed informed consent prior to participation.

The study included two groups: an ortho-k group and a healthy control group. The healthy control group corresponded to subjects previously evaluated by our research group using the same experimental protocol and instrumentation [10]. The inclusion criteria for the ortho-k group comprised subjects who had undergone overnight ortho-k treatment for at least two weeks and were older than 18 years. The healthy control group included participants older than 18 years who met the same inclusion and exclusion criteria, except for ortho-k treatment. Subjects with ocular pathology, such as corneal ectasia (e.g., keratoconus), or a history of ocular surgery were excluded.

### 2.2. Sample Size Calculation

Sample size calculation was performed using the online GRANMO calculator version 8.0 (IMIM, Barcelona, Spain) to determine the minimum number of eyes required to detect significant changes in corneal parameters throughout the day. As no previous studies have evaluated circadian changes in ortho-k wearers, the calculation was based on the study by Çiçek et al. [11], which investigated diurnal variations in anterior segment parameters in patients with keratoconus. Based on the reported changes in central corneal thickness, and assuming a minimum detectable difference of 0.0148 mm, a common standard deviation of 0.008 mm, an anticipated dropout rate of 20%, a statistical power of 99.9%, and a significance level (α) of 0.05, the minimum required sample size was estimated to be 19 eyes.

The healthy control group included 55 right eyes from 55 participants, corresponding to a previously published dataset obtained using the same study protocol [10].

### 2.3. Experimental Procedure and Instrumentation

Each participant underwent a comprehensive ocular assessment carried out by a single experienced examiner (L.B.B.) to ensure measurement consistency. The baseline evaluation included medical history collection, subjective and objective refraction, and slit-lamp examination.

To investigate intra-day variability, all parameters were recorded repeatedly at five intervals distributed across the day. Data acquisition started in the morning (9:00 a.m.) and continued every 2.5 h until the evening (7:00 p.m.), allowing five assessments of diurnal patterns over a ten-hour span. The same examination protocol, measurement schedule, and instrumentation were applied to both groups.

Corneal characteristics, such as corneal thickness and anterior and posterior keratometry, were evaluated with Pentacam tomography (OCULUS Optikgeräte GmbH, Wetzlar, Germany). Autorefractometry was assessed using the Visionix 650 multidiagnostic platform (Visionix, Chartres, France). Visual acuity was measured using an ETDRS (Early Treatment Diabetic Retinopathy Study) optotype.

### 2.4. Corneal Surface Reconstruction and Geometric Analysis

Anterior corneal elevation data obtained from corneal topography were reconstructed as continuous analytical surfaces using Zernike polynomial expansions. Elevation maps were fitted with Zernike polynomials up to the seventh order, resulting in smooth and twice-differentiable surfaces suitable for analytical computation of spatial derivatives [12].

Local corneal geometry was characterized using mean curvature (*κ_H_*, [m^−1^]), an intrinsic descriptor derived from the two principal curvatures of the surface and independent of the selected coordinate system or reference axis [13]. In this study, the term mean curvature refers to mean optical power (H, in diopters). Beyond its geometric interpretation, corneal curvature is mechanically associated with the balance between intraocular pressure and membrane stress under thin-shell approximations [14,15], and may therefore indirectly reflect variations in corneal mechanical behavior.

(1) $$\kappa_{H}\left(x,y\right)=\frac{\left(1+z_{y}^{2}\right)z_{xx}-2z_{x}z_{y}z_{xy}+\left(1+z_{x}^{2}\right)z_{yy}}{2{\left(1+z_{x}^{2}+z_{y}^{2}\right)}^{3/2}}$$

where (*z_x_*, *z_y_*) and (*z_xx_*, *z_xy_*, *z_yy_*) denote the first and second-order derivatives of the reconstructed surface.

Curvature values were subsequently converted into optical power assuming a spherical refractive interface. For the anterior corneal surface, refractive indices of air (*n*_0_ = 1.000) and cornea (*n*_1_ = 1.376) were considered, whereas for the posterior surface the refractive indices of cornea (*n*_0_ = 1.376) and aqueous humor (*n*_1_ = 1.336) were used [16]. Local corneal power was calculated as:

(2) $$H\left(x,y\right)=\left(n_{1}-n_{0}\right)\kappa_{H}\left(x,y\right)\;\left[D\right]$$

### 2.5. Regional and Statistical Analysis

To evaluate spatial variations in corneal geometry, the cornea was partitioned into concentric annular regions centered on the corneal apex with radii of 0–1, 1–2, 2–3, and 3–4 mm. The central annulus (0–1 mm) was analyzed as a single region, whereas the peripheral annuli were further subdivided into nasal-superior, temporal-superior, temporal-inferior, and nasal-inferior sectors. Mean annular values were additionally computed for each peripheral ring. For each eye, surface, visit, ring, and sector, average values of corneal thickness (T) and mean corneal power (H) were extracted from the corresponding maps.

### 2.6. Data Analysis

All statistical procedures were performed using SPSS (version 29.0.1; IBM Corp., Chicago, IL, USA). Baseline demographic and clinical characteristics were compared between the healthy control and ortho-k groups using the Mann–Whitney U test for continuous variables and Fisher’s exact test for categorical variables. The amplitude of diurnal variation was calculated for each parameter as the difference between the maximum and minimum values recorded across the five measurement time points. Amplitude values were compared between the healthy control and ortho-k groups using the Mann–Whitney U test. The Friedman test for repeated measures was applied to detect differences across time points [17]. When significant effects were observed, Bonferroni-adjusted post hoc pairwise comparisons were performed to control for type I error associated with multiple testing. Statistical significance was set at *p* < 0.05.

## 3. Results

### 3.1. Sample Characteristics

Nineteen eyes from 10 ortho-k wearers (9 right eyes and 10 left eyes) were included in the study. A healthy control group consisting of 55 right eyes from 55 participants from our previous study [10] was included for comparative analyses. The baseline characteristics of both groups are summarized in Table 1. The mean pre-treatment spherical equivalent was −3.74 ± 1.25 D. All participants wore the same ortho-k lens design (Alexa, Tiedra Farmacéutica, Madrid, Spain) and had worn the lenses for a median of 27.5 days (IQR: 25.0–52.5 days) prior to assessment.

### 3.2. Diurnal Changes in Ortho-k Wearers

Table 2 summarizes the diurnal evolution of the clinical and corneal parameters analyzed across the five measurement sessions.

To determine whether the observed changes exceeded normal physiological diurnal variations, the amplitudes of corneal thickness and curvature changes were compared between the ortho-k and healthy control groups in Table 3. Ortho-k wearers showed significantly greater diurnal amplitudes of CCT (*p* = 0.019), anterior K1 (*p* < 0.001), and anterior K2 (*p* < 0.001), whereas no significant differences were found for MCT, posterior corneal curvature, or corneal asphericity.

**Figure 1:**
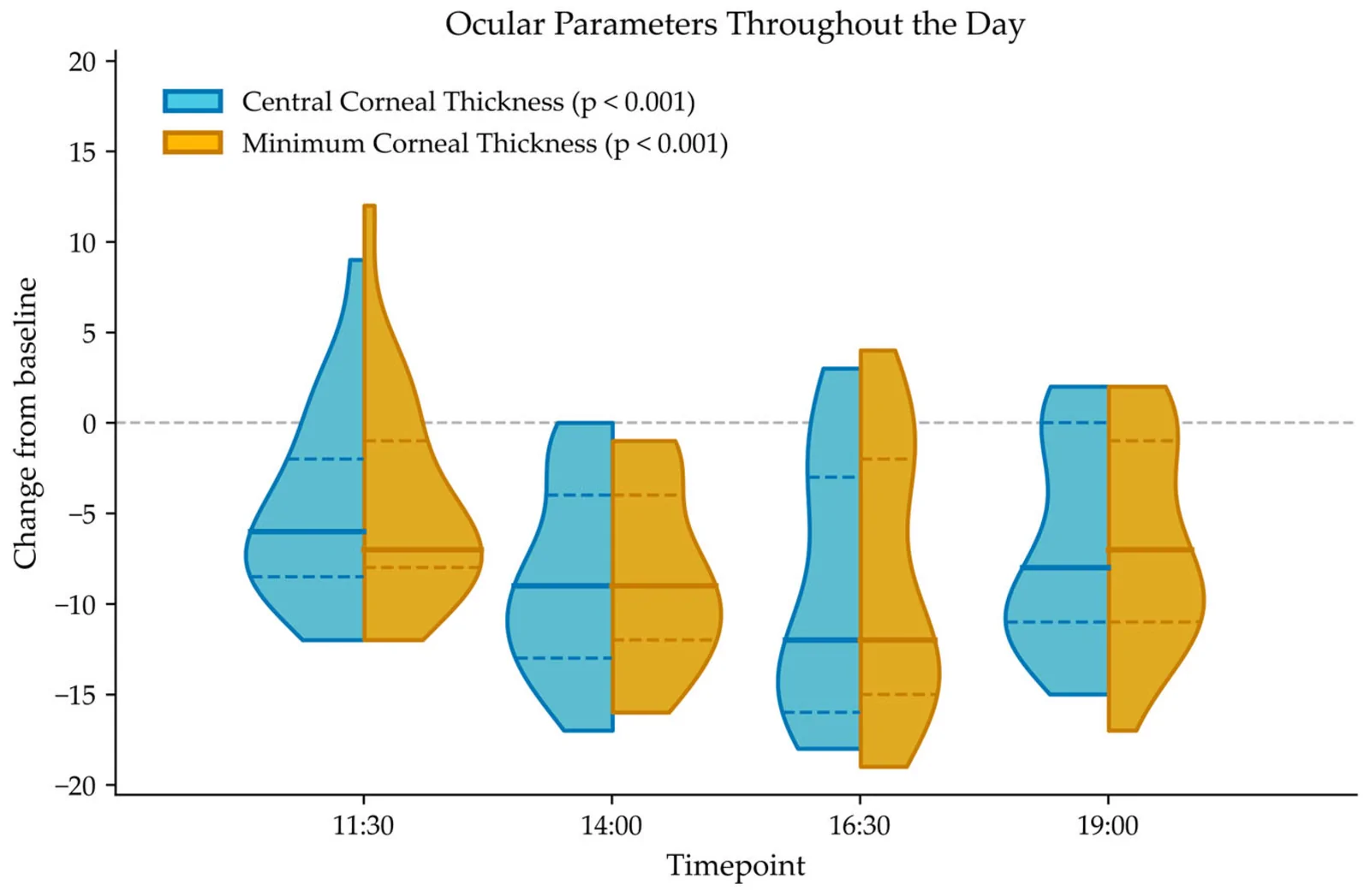
Relative changes throughout the day from baseline in corneal thickness. The solid line within each violin plot represents the median, while the dashed lines indicate the interquartile range (25th and 75th percentiles). The *p*-values shown in the legend correspond to the Friedman repeated-measures test across the five measurement time points.

Corneal thickness progressively decreased throughout the day. Post hoc Bonferroni analysis revealed significantly lower central thickness (CCT) and minimum corneal thickness (MCT) values at visits 3, 4, and 5 compared with the initial morning measurement (*p* ≤ 0.002), as well as between visits 2 and 3 (*p* ≤ 0.004). The changes from baseline for corneal thickness are depicted in Figure 1.

**Figure 2:**
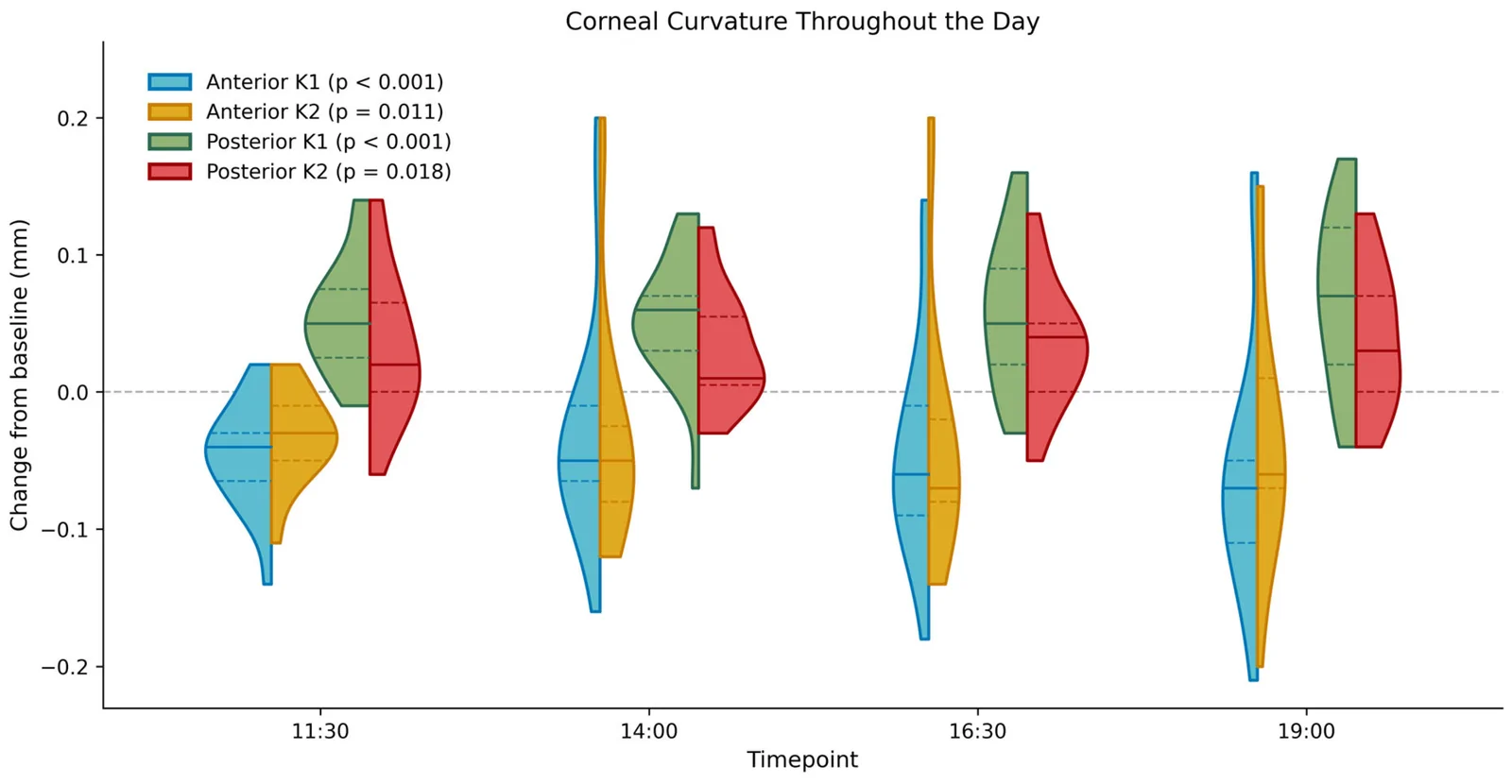
Relative changes throughout the day from baseline in anterior and posterior corneal curvature. The solid line within each violin plot represents the median, while the dashed lines indicate the interquartile range (25th and 75th percentiles). The *p*-values shown in the legend correspond to the Friedman repeated-measures test across the five measurement time points.

Anterior keratometric parameters showed a gradual increase in corneal curvature throughout the day. Significant differences in anterior flattest keratometry (K1) were detected between the first and second visit (*p* = 0.001), and between the third and fifth visit (*p* = 0.003), whereas anterior steepest keratometry (K2) differed significantly only between visits 1 and 2 (*p* = 0.004). Posterior corneal curvature also demonstrated temporal variations. Posterior K1 differed significantly between the first visit and visits 2, 3, 4, and 5 (all *p* ≤ 0.003), while posterior K2 showed significant differences only between visits 1 and 3 (*p* = 0.004). The variation from the initial measurement in corneal curvature throughout the day is shown in Figure 2.

**Figure 3:**
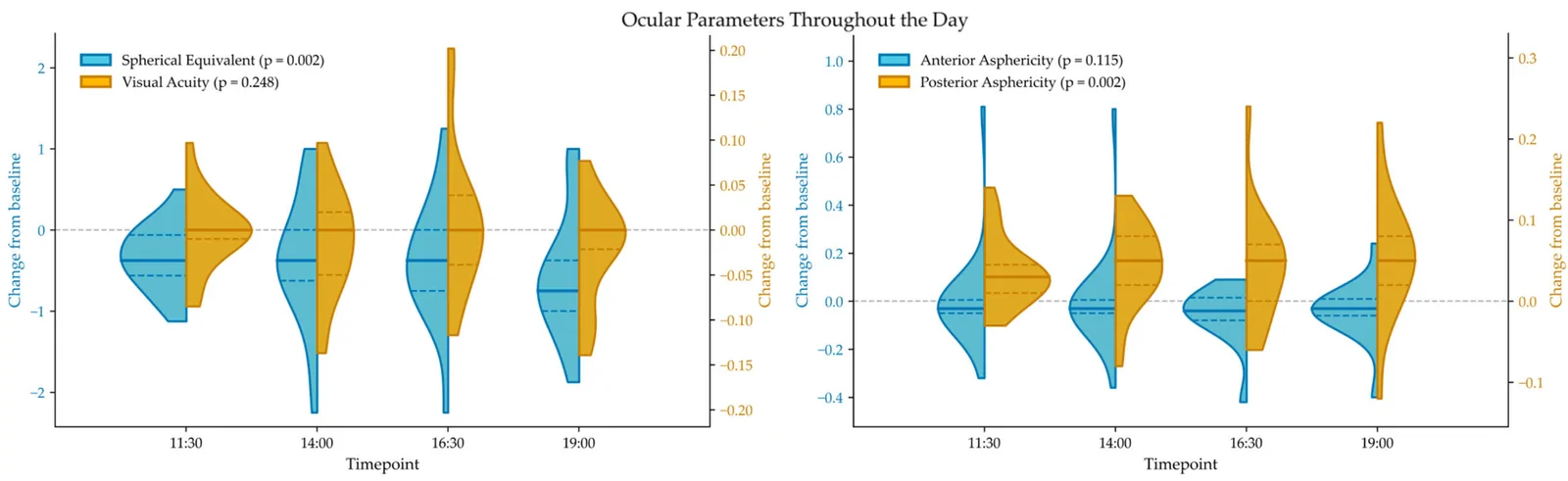
Relative changes throughout the day from baseline in refractive error, visual acuity and asphericity. The solid line within each violin plot represents the median, while the dashed lines indicate the interquartile range (25th and 75th percentiles). The *p*-values shown in the legends correspond to the Friedman repeated-measures test across the five measurement time points.

Autorefractometry measurements revealed a progressive myopic shift during the day, with significant differences in spherical equivalent between visits 1 and 2 (*p* = 0.003). Regarding corneal asphericity, posterior asphericity showed significant differences between the first visit and visits 2 and 3 (*p* = 0.003 and *p* = 0.002, respectively). Figure 3 illustrates the relative variation from baseline for refractive error, visual acuity and asphericity throughout the day.

**Figure 4:**
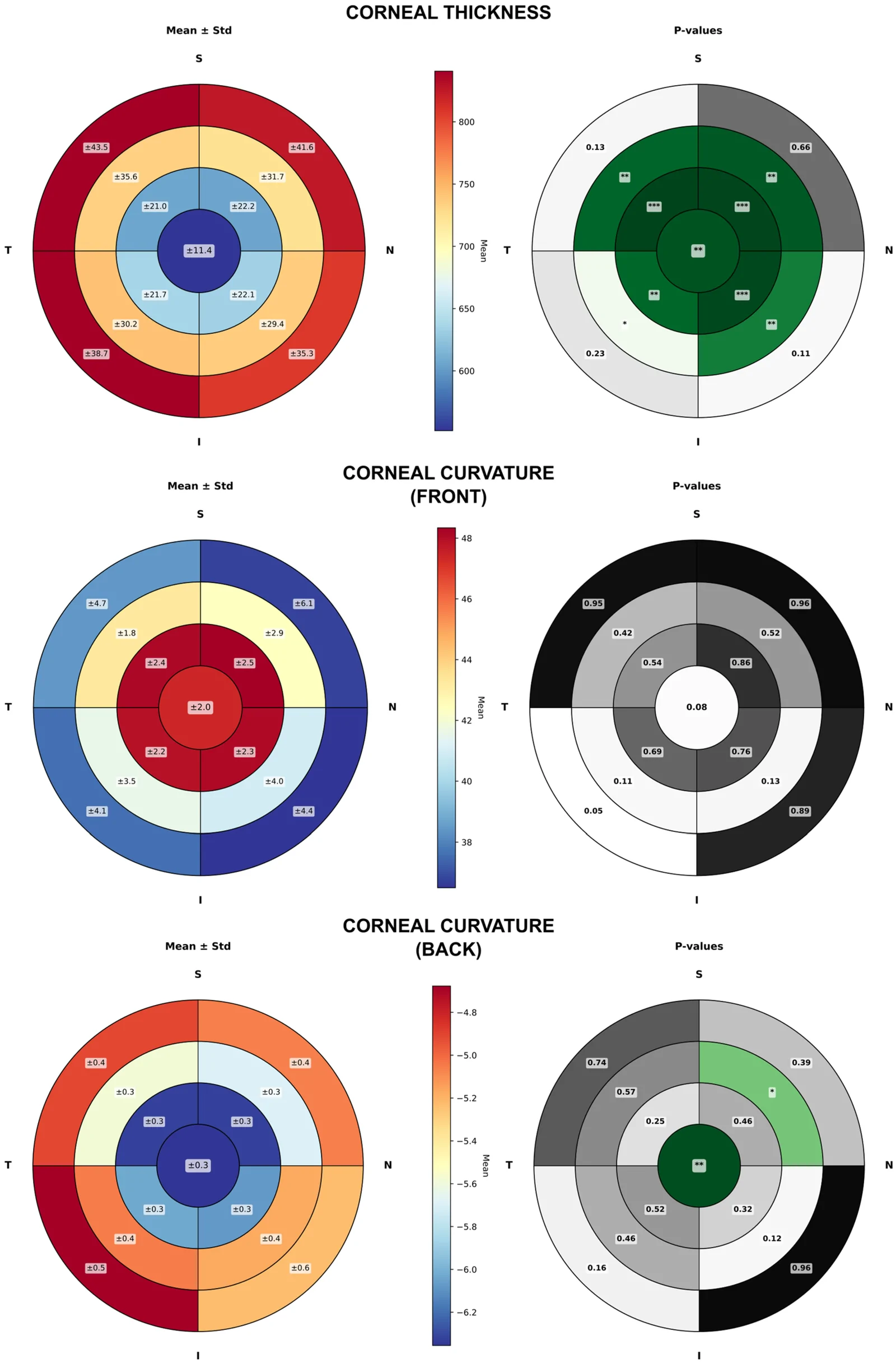
Sector-based analysis of diurnal changes in corneal thickness and anterior and posterior corneal curvature. Mean ± SD maps (left) and corresponding *p*-value maps (right) are shown, with green indicating significant changes and grey indicating non-significant regions. Each concentric ring has a width of 1 mm. Statistical significance is indicated as * *p* < 0.05, ** *p* < 0.01, and *** *p* < 0.001. Green shades indicate regions with statistically significant changes, with darker tones corresponding to lower *p*-values. Grey shades represent non-significant regions, with darker tones corresponding to higher *p*-values.

Figure 4 shows a sector-based plot illustrating the diurnal variation of CCT and anterior and posterior corneal curvature across different corneal regions. A general decrease in CCT over the course of the day is observed across most sectors, indicating a consistent temporal trend. In contrast, anterior corneal curvature remains largely stable throughout the day across sectors, with no statistically significant sectorial changes. The central 2 mm region shows a borderline effect (*p* = 0.08), although this does not reach statistical significance. Regarding posterior corneal curvature, significant flattening is observed in the central corneal region, suggesting a diurnal modulation of posterior corneal shape predominantly affecting the central area (*p* < 0.01).

## 4. Discussion

The progressive decrease in corneal thickness observed throughout the day likely reflects the physiological recovery from overnight corneal edema [18]. Several studies have demonstrated that corneal thickness progressively decreases during the day in healthy corneas [10,19] and in several conditions such as pseudoexfoliation or corneal dystrophy [20,21,22]. Therefore, it remains unclear whether the reduction observed in the present study is exclusively related to ortho-k treatment or partially reflects normal circadian variations in corneal hydration and thickness. However, a previous study with the same methodology conducted by our research group in healthy subjects reported a mean diurnal amplitude of 8.24 µm and 8.38 µm for CCT and MCT, respectively [10]. In contrast, the present study found larger amplitudes of 11.74 µm for CCT and 11.42 µm for MCT. These amplitudes were substantially greater than the measurement variability reported for the Pentacam HR in healthy eyes, which is generally below 5 µm [23], suggesting that the observed changes are unlikely to be explained entirely by instrument repeatability and probably represent true physiological variations. Although corneal thickness changes were greater in ortho-k wearers than in healthy subjects, they remained substantially smaller than the overnight corneal swelling observed in pathological conditions such as diabetes mellitus, which is approximately 50–60 µm [24]. These findings are further supported by comparison with the healthy control group, which showed significantly greater amplitudes of CCT in ortho-k wearers. Together, these results suggest that ortho-k treatment enhances the normal physiological diurnal fluctuations in corneal thickness, potentially due to the combined effects of overnight lens wear, epithelial redistribution, and altered stromal hydration dynamics. This is an aspect that should be investigated further in future studies.

A progressive steepening was observed in the anterior corneal surface between the first measurement obtained after awakening and the assessment performed 2.5 h later, with an amplitude of 0.11 mm in radius (*p* ≤ 0.011). However, sector-based analysis did not reveal localized regional changes. The magnitude of change was greater than previously reported in healthy corneas, where diurnal variations ranged between 0.03 and 0.07 mm in radius [25,26]. Similarly, a previous study conducted by our research group found a mean anterior corneal power amplitude of 0.8 D, corresponding to approximately 0.04 mm in radius [10]. The observed change corresponds to an approximate keratometric variation of 0.6 D, which exceeds the reported repeatability limits of the instrument in healthy eyes (<0.24 D) [23,27], suggesting that the observed changes do not appear to be explained by measurement variability alone. The larger changes observed in ortho-k subjects may result from the combination of normal circadian corneal fluctuations and the progressive regression of the ortho-k-induced flattening effect throughout the day. Consistent with this hypothesis, the amplitudes of anterior K1 and K2 were significantly greater in ortho-k wearers than in healthy subjects, indicating that ortho-k amplifies the normal diurnal changes in anterior corneal curvature (*p*-values < 0.001).

In contrast, the posterior corneal surface showed progressive flattening after the first morning measurement, with an amplitude of 0.10 mm (*p* ≤ 0.018). Sectorial analysis revealed that this effect was mainly localized within the central 2 mm corneal region. In our previous study in healthy corneas, posterior corneal curvature did not exhibit significant circadian variations [10], suggesting that the changes observed in the present study may be predominantly associated with the ortho-k effect. Although the overall amplitude of posterior corneal curvature changes did not differ significantly between the two groups, ortho-k wearers exhibited a more consistent and progressive trend throughout the day, whereas healthy subjects showed a more variable pattern of physiological fluctuations. Nevertheless, Read and Collins reported a slight posterior corneal steepening of approximately 0.04–0.05 D throughout the day in healthy subjects [28], indicating that small physiological diurnal fluctuations in posterior corneal geometry cannot be completely excluded.

Several authors found that posterior corneal curvature remains largely stable during ortho-k treatment [29,30,31]. Likewise, Yoon and Swarbrick observed no clinically relevant posterior curvature changes, although an increase in posterior corneal asphericity values was detected during the first week of treatment [32]. However, some studies have suggested that subtle posterior alterations may occur, particularly during the early adaptation phase or after long-term wear. Owens et al. reported significant early posterior surface flattening [33], while González-Mesa et al. described sector-dependent posterior corneal flattening after both short and long-term ortho-k treatment [34]. Overall, the current literature indicates that posterior corneal changes associated with ortho-k are generally smaller and less consistent than anterior changes, although transient or localized modifications cannot be ruled out.

Ortho-k lenses act on the epithelium and anterior corneal stroma [35], without directly inducing structural changes in the posterior corneal surface. Therefore, the refractive effects observed after treatment cannot be fully explained by anterior surface reshaping alone. These changes may be associated with modifications in the effective corneal refractive index, potentially driven by alterations in the spatial distribution, hydration state, and organization of stromal collagen fibers during ortho-k wear. In this context, Barbero developed a model of the cornea showing that the contribution of stromal redistribution to the overall refractive power is relatively limited. Specifically, the model predicted a paraxial refractive change of only 0.36 D, whereas the clinically measured refractive change in ortho-k reached 2.63 D [36].

Visual acuity remained stable throughout the day, indicating that the ortho-k effect was sufficient to maintain functional visual correction despite the progressive geometric and refractive changes observed after lens removal. Johnson et al. reported a trend toward worse visual acuity throughout the day in ortho-k wearers, although statistically significant differences were only observed under high-contrast and low-luminance optotype conditions [37].

Refractive measurements revealed a progressive myopic shift of 1.03 D over the 10-h observation period, confirming the gradual regression of the corneal reshaping effect during the day [38]. However, this myopic shift did not affect visual acuity, as ortho-k treatment typically induces a slight hyperopic overcorrection. As the treatment effect gradually regresses throughout the day, this initial hyperopic shift decreases, partially compensating for the progressive myopic change and helping to maintain stable visual acuity. Similar daytime refractive regression has previously been described in ortho-k wearers [37] and can be considered part of the normal evolution of the treatment effect after overnight lens wear. Another study reported a difference of approximately one diopter between morning and afternoon measurements after two weeks of ortho-k treatment, although refractive stability improved after 12 weeks of treatment [9].

Regarding corneal asphericity, no significant diurnal changes were observed in anterior Q values, suggesting relative stability of the overall anterior corneal profile throughout the day. The observed changes in posterior corneal Q were consistent with the keratometric findings. Specifically, posterior Q values showed significant diurnal variations, becoming less negative after the first morning measurement. Yoon and Swarbrick reported a significant increase toward a more oblate anterior corneal Q during the early phase of ortho-k treatment, whereas posterior Q changes were smaller and transient, becoming not statistically significant after two weeks of lens wear [32].

Patients in this study wore ortho-k lenses for a minimum of two weeks. However, Kang et al. reported that the differences between morning and afternoon stabilized by the twelfth week of treatment [9]. Although participants presented good visual acuity, it is possible that some had not yet achieved complete refractive and corneal stabilization, which may represent a limitation of the present study. Therefore, the results should be interpreted with caution, and future studies should preferably include only long-term ortho-k wearers with fully stabilized treatment effects.

Another limitation is that no direct comparison was performed with a healthy control group. The comparisons presented here are based on the results of a previous study from our research group that used the same protocol in healthy eyes [10]. Future studies should directly compare healthy subjects and ortho-k wearers to determine whether the differences observed between both populations are statistically significant. In addition, the relatively small sample size may have reduced the statistical power to detect subtle regional or temporal changes, but it should be considered as a preliminary analysis confirming that these aspects deserve further research. The limited sample size also did not allow meaningful subgroup analyses according to factors such as lens wearing time, sleep duration, or baseline myopic refractive error. Grouping participants into these variables, despite the potential influence of these factors on diurnal changes, would have resulted in very small groups and insufficient statistical power. A larger cohort would improve the robustness and generalizability of the findings.

In conclusion, ortho-k wearers exhibited significant diurnal changes in corneal geometry, including progressive thinning of the cornea, anterior corneal steepening, posterior central flattening, and a gradual myopic refractive shift throughout the day. These variations were greater than those previously reported in healthy corneas, suggesting that ortho-k may enhance normal circadian corneal fluctuations. Despite these geometric and refractive changes, visual acuity remained stable during the evaluated period, indicating that functional visual correction was maintained. The findings of the present study highlight the importance of considering measurement timing during ortho-k follow-up and contribute to a better understanding of the temporal behavior of corneal reshaping induced by ortho-k.

## Figures and Tables

**Table 1:** Baseline demographic and ocular characteristics of the ortho-k and healthy control groups.

Variable	Ortho-k	Controls	*p*-Value
Age (years)	25.60 ± 5.40	32.55 ± 12.60	0.167
Sex, n (female/male)	7/3	37/18	1.000
Sleep duration (h)	7.51 ± 0.71	7.14 ± 1.04	0.240
Time since awakening (h)	1.60 ± 0.57	2.08 ± 0.83	0.076
Axial length (mm)	25.01 ± 0.85	23.89 ± 1.13	<0.001 *
Visual acuity (logMAR)	−0.01 ± 0.10	−0.10 ± 0.06	<0.001 *

Participant characteristics are presented per participant, whereas ocular characteristics are presented per eye. Data are presented as mean ± SD unless otherwise indicated. *p*-values were obtained using the Mann–Whitney U test for continuous variables and Fisher’s exact test for sex. * indicates statistically significant *p*-values (*p* < 0.05).

**Table 2:** Corneal and refractive changes throughout the day.

Parameter	9:00	11:30	14:00	16:30	19:00	*p*-Value
CCT (µm)	538.47 ± 13.16	533.84 ± 9.83	529.89 ± 10.84	527.00 ± 8.82	529.24 ± 9.63	<0.001 *
MCT (µm)	534.16 ± 13.03	529.84 ± 9.67	525.68 ± 11.36	523.29 ± 8.90	525.24 ± 9.84	<0.001 *
Anterior K1 (mm)	8.27 ± 0.31	8.22 ± 0.32	8.24 ± 0.35	8.26 ± 0.34	8.24 ± 0.36	<0.001 *
Anterior K2 (mm)	8.05 ± 0.35	8.02 ± 0.40	8.02 ± 0.41	8.04 ± 0.42	8.04 ± 0.42	0.011 *
Posterior K1 (mm)	6.46 ± 0.33	6.52 ± 0.32	6.52 ± 0.33	6.54 ± 0.33	6.55 ± 0.33	<0.001 *
Posterior K2 (mm)	6.17 ± 0.36	6.21 ± 0.37	6.20 ± 0.36	6.23 ± 0.38	6.23 ± 0.38	0.018 *
Anterior Q	−0.04 ± 0.23	−0.04 ± 0.09	−0.04 ± 0.09	−0.05 ± 0.07	−0.08 ± 0.16	0.115
Posterior Q	−0.30 ± 0.08	−0.26 ± 0.07	−0.25 ± 0.09	−0.27 ± 0.09	−0.25 ± 0.11	0.002 *
SER (D)	−0.11 ± 1.02	−0.45 ± 0.98	−0.46 ± 1.10	−0.51 ± 0.93	−0.68 ± 0.99	0.002 *
VA (logMar)	−0.01 ± 0.10	−0.01 ± 0.12	−0.03 ± 0.12	0.00 ± 0.15	−0.03 ± 0.12	0.248

Data are presented as mean ± standard deviation (SD). *p*-values correspond to the Friedman test for comparisons across the five time points. * indicates statistically significant *p*-values (*p* < 0.05). Abbreviations: CCT = central corneal thickness; MCT = minimum corneal thickness; Q = asphericity (dimensionless); SER = spherical equivalent refraction; VA = visual acuity.

**Table 3:** Comparison of the diurnal amplitudes between ortho-k wearers and healthy controls.

Parameter	Ortho-k	Controls	Difference	*p*-Value
CCT (µm)	11.74 ± 5.60	8.29 ± 4.24	3.45 ± 6.97	0.019 *
MCT (µm)	11.42 ± 6.10	9.04 ± 6.75	2.38 ± 9.10	0.053
Anterior K1 (mm)	0.10 ± 0.05	0.05 ± 0.04	0.06 ± 0.06	<0.001 *
Anterior K2 (mm)	0.10 ± 0.06	0.05 ± 0.02	0.05 ± 0.06	<0.001 *
Posterior K1 (mm)	0.10 ± 0.04	0.10 ± 0.04	0.01 ± 0.05	0.392
Posterior K2 (mm)	0.09 ± 0.03	0.09 ± 0.04	0.00 ± 0.05	0.669

Data are presented as mean ± standard deviation (SD). Difference represents the mean difference in diurnal amplitude between the ortho-k and healthy control groups. *p*-values were obtained using the Mann–Whitney U test. * indicates statistically significant differences (*p* < 0.05). Abbreviations: CCT = central corneal thickness; MCT = minimum corneal thickness.

## Data Availability

Data available on request from the authors.

## Abbreviations

The following abbreviations are used in this manuscript:

Ortho-k	Orthokeratology
CCT	Central Corneal Thickness
MCT	Minimum Corneal Thickness
K1	Flattest Keratometry
K2	Steepest Keratometry
